# Evaluation of Risk Factors Associated With Adult-Onset Acne in Patients Attending a Tertiary Care Center in East India: A Case-Control Study

**DOI:** 10.7759/cureus.53296

**Published:** 2024-01-31

**Authors:** Surbhi Kashyap, Laxman Besra, Hemanta K Kar

**Affiliations:** 1 Department of Dermatology, Venereology and Leprosy, Kalinga Institute of Medical Sciences, Bhubaneswar, IND

**Keywords:** cosmetics, stress, hormones, risk factor, adult-onset acne

## Abstract

Background: In recent years, the aesthetic appearance of the skin has emerged as a crucial factor influencing perceptions of beauty and contributing to self-confidence. The pursuit of flawless skin represents a prevalent focus within beauty regimens. Adult-onset acne (AOA) is the development of acne between the ages of 26 to 50 and it is emerging as a prevalent dermatological concern among this population. Individuals perceiving their skin as falling short of an 'ideal' standard may let it affect their quality of life. Significant gaps in our understanding persist regarding the contributing risk factors for AOA.

Objective: The study aims to assess both established and novel risk factors potentially influencing the onset of adult acne. Additionally, it seeks to calculate the odds ratio (OR) for AOA in both females and males exposed to the surveyed risk factors over a 24-month period.

Materials and methods: Various risk factors were assessed, including stress, hormonal markers, psychological factors, environmental exposures, dietary habits, and cosmetic use. A total of 140 participants, consisting of 70 healthy individuals were selected. Discordant groups were analyzed for AOA. Detailed interviews were conducted to obtain a comprehensive medical history, focusing on potential risk factors, for patients diagnosed with acne. The OR was calculated to determine the likelihood of association between risk factors and the development of AOA. A proper protocol was devised, and statistical data was analyzed using Statistical Package for Social Sciences (SPSS; IBM Corp., Armonk, NY, USA).

Results: The most significant risk factors in the development of AOA in the Indian population based on OR and confidence interval (CI) were positive personal history of acne (OR 3.12 [95% CI 1.20 - 8.03]), positive family history of acne (OR 10.24 [95% CI 2.89 - 36.1]), overweight BMI (OR 6.16 [95% CI 2.56 - 14.76]), hormonal imbalance (OR 9.27 [95% CI 2.03 - 42.29]), menstrual irregularity in females (OR 12.94 [95% CI 3.59 - 46.53]), exposure to mineral oil or halogenated hydrocarbon use (OR 4.13 [95% CI 1.28 - 13.24]), less than six hours of sleep (OR 4.16 [95% CI 1.10 - 15.64]), chemical peels in females (OR 11.28 [95% CI 2.45 - 51.90]), diet consisting mainly of carbohydrates, high salt, saturated fats (OR 29.97 [95% CI 3.84 - 227.25]) and less than 2 liters of water intake in patients (OR 19.18 [95% CI 1.08 - 339.04]). Risk factors that were associated with a decreased likelihood of AOA included normal menstruation (OR 0.03 [95% CI 0.01 - 0.12]), healthy oral intake (OR 0.04 [95% CI 0.00 - 0.17]), no psychological stressors/depression/anxiety (OR 0.43 [95% CI 0.21 - 0.85]), no environmental factors (OR 0.07 [95% CI 0.02 - 0.24]), no associated cosmetic use (OR 0.45 [95% CI 0.22 - 0.90]), normal BMI (OR 0.18 [95% CI 0.07 - 0.39]), no history of acne (OR 0.12 [95% CI 0.05 - 0.26]).

Conclusion: AOA is a complex and multifactorial condition, and most of the risk factors mentioned in this study on Indian skin type contribute to its development. The approach for AOA should be holistic. In addition to following a recommended treatment protocol, education should be provided about lifestyle modification, stress management, exercise, and environmental factors to help prevent and manage AOA.

## Introduction

Acne vulgaris is a chronic inflammatory skin disease of pilosebaceous units that manifests with polymorphic lesions and can be graded based on appearance of comedones, papules, pustules, nodules and cysts. It is one of the most prevalent skin conditions affecting more than 85% of adolescents worldwide [1]. Although acne is commonly associated with adolescents, there has been an increase in cases of adult-onset acne (AOA). Adult acne is categorized as acne that develops after age 25 and is divided into persistent and late-onset acne. If adolescent acne persists beyond age 25, it is called persistent adult acne [2].

In previous studies, AOA has been linked to several causes, including genetic predisposition, hormonal influences (role of androgens, 5α reductase activity, polycystic ovarian disease), dietary factors, stress, cigarette smoking, and cosmetic items containing comedogenic substances such as lanolin, petrolatum, and vegetable oils. The prevalence of psychiatric comorbidity in adult acne patients has been documented up to 40% [3]. However, very few studies have been conducted on the occurrence and risk factors associated with acne in adults, particularly among the Indian population. This study aims to determine the odds ratio (OR) of AOA in females and males exposed to the surveyed risk factors over two years.

## Materials and methods

Study overview

This prospective case-control study spanned 24 months, from September 2019 to August 2021. The investigation aimed to assess the association between various risk factors and AOA. The study involved 70 patients with AOA and 70 healthy controls without acne, recruited from the outpatient Dermatology Department at a tertiary care center in Bhubaneswar, Odisha, India.

Ethical considerations

Ethical approval was obtained from the institutional ethical committee [KIIT/KIMS/IEC/91/2019] at Kalinga Institute of Medical Sciences, Bhubaneswar, before the commencement of the study. The study adhered to ethical standards, ensuring participant confidentiality, welfare, and compliance with relevant regulations.

Study population

The study included adults aged 25-50 with varying grades of acne. Exclusion criteria comprised individuals taking oral contraceptive pills or undergoing hormonal therapy. The case group consisted of 70 patients with AOA, while 70 healthy individuals without AOA served as the control group.

Study procedure

Data collection involved a detailed history evaluation, dermatological examination, and psychological assessment. Discordant groups were analyzed for AOA, and risk factors were assessed using various parameters, including age of onset, occupation, disease duration, lifestyle factors, and relevant medical history. Risk factors that were significant in the development of AOA were analyzed and the OR and confidence interval (CI) for each risk factor was calculated in females and males. A detailed history was obtained to evaluate the relevant risk factors for AOA, including the following: age of onset, sex, occupation, duration of the disease, frequency of eruptions, exposure to risk factors, aggravating factors, body mass index (BMI), application of cosmetics, topical steroid use, drug history, stress, sleep, menstrual history, seasonal variation, photo-sensitivity, diet, exercise, markers of androgenicity, alcohol intake, cigarette use, gynecological history, past medical and family history. Patients with irregular menstrual history were advised blood investigations: complete blood count (CBC), liver function test (LFT), fasting blood sugar, hemoglobin A1c (HbA1c), insulin resistance testing, luteinizing hormone (LH), follicle-stimulating hormone (FSH), estrogen, testosterone, cortisol levels, dehydroepiandrosterone (DHEA), abdominal and pelvic ultrasound, to rule out hormonal causes and endocrine disorders associated with acne.

Assessments

A comprehensive dermatological examination was conducted, documenting acne lesions, Fitzpatrick skin type, and grading of acne. Psychological factors were assessed using Patient Health Questionnaire 9 (PHQ-9) and General Anxiety Disorder 7 (GAD-7) assessments.

Statistical analysis

Statistical analysis utilized Statistical Package for Social Sciences (SPSS) version 24 (IBM Corp., Armonk, NY, USA) software, with data tabulated in Microsoft Excel 2016. Continuous and categorical variables were analyzed using Pearson chi-square and Fisher’s exact test, determining the OR, 95% CI, and statistical significance for associated risk factors.

## Results

During the study period, 112 patients were evaluated for inclusion, of which 70 patients with AOA fulfilled the criteria. Seventy healthy controls were selected, for a total of 140 participants. The mean age in years for the case group was 30.5 ± 5.08. The mean age in years for the control group was 28.7 ± 3.55. The OR, CI, and significance of the surveyed risk factors and their association with AOA were calculated and listed (Table 1).

**Table 1:** Odds ratio of associated risk factors and their correlation with adult-onset acne The data is split into three columns: female, male and combined. AOA = Adult-Onset Acne, BMI = Body Mass Index, Carb = Carbohydrate, L = Liters, PCOS = Polycystic Ovarian Syndrome, CI = Confidence Interval

	Female						Male						Total				
Risk Factors	AOA	No AOA	Odds Ratio	95% CI	P		AOA	No AOA	Odds Ratio	95% CI	P		AOA	No AOA	Odds Ratio	95% CI	P
Acne History																	
No History of Acne	27	51	0.11	(0.04 - 0.30)	<0.001		2	9	0.07	(0.01 - 0.45)	0.006		29	60	0.12	(0.05 - 0.26)	<0.001
Personal History	15	5	3.90	(1.30 - 11.63)	0.015		3	2	1.38	(0.19 - 9.83)	0.751		18	7	3.12	(1.20 - 8.03)	0.019
Family History	13	1	17.33	(2.18 - 137.77)	0.007		9	2	8.25	(1.32 - 51.26)	0.024		22	3	10.24	(2.89 - 36.15)	<0.001
Personal and Family History	0	0	1.04	(0.02 - 53.12)	0.986		1	0	2.79	(0.10 - 74.62)	0.540		1	0	3.04	(0.12 - 75.99)	0.498
Body Mass Index																	
Under weight BMI <18.5	1	2	0.51	(0.04 - 5.78)	0.586		0	0	0.87	(0.01 - 46.95)	0.946		1	2	0.49	(0.04 - 5.56)	0.567
Normal BMI 18.5-24.9	30	47	0.26	(0.10 - 0.60)	0.002		4	12	0.03	(0.00 - 0.31)	0.003		34	59	0.18	(0.07 - 0.39)	< 0.0001
Over weight BMI 25-29.9	23	8	4.40	(1.75 - 11.04)	0.002		8	0	30.60	(1.54 - 607.67)	0.025		31	8	6.16	(2.56 - 14.76)	< 0.0001
Obese BMI >30	1	0	3.17	(0.12 - 79.37)	0.483		3	1	3.00	(0.27 - 33.08)	0.370		4	1	4.18	90.45 - 38.39)	0.206
Cosmetics																	
No Associated Cosmetics	13	23	0.46	(0.20 - 1.03)	0.061		8	11	0.21	(0.03 - 1.27)	0.090		21	34	0.45	(0.22 - 0.90)	0.026
Sunscreen Use	0	3	0.14	(0.01 - 2.78)	0.197		1	0	2.79	(0.10 - 74.62)	0.540		1	3	0.32	(0.03 - 3.19)	0.334
Facewash/Cream/Lotion Use	9	19	0.39	(0.15 - 0.96)	0.042		3	1	3.00	(0.27 - 33.08)	0.370		12	20	0.5172	(0.23 - 1.16)	0.110
Peels/Facial	16	2	11.28	(2.45 - 51.90)	0.002		1	0	2.79	(0.10 - 74.62)	0.540		17	2	0.99	(0.47 - 2.05)	0.982
Ayurvedic Products	17	10	2.10	(0.86 - 5.12)	0.102		2	1	1.85	(0.14 - 23.07)	0.634		19	11	2.00	(0.86 - 4.59)	0.103
Environmental Factors																	
Sweating/Humidity/Elevated Temperatures	11	4	3.31	(0.98 - 11.13)	0.053		9	4	5.00	(0.95 - 26.10)	0.056		20	8	3.10	(1.25 - 7.62)	0.014
Sun Exposure	5	3	1.80	(0.40 - 7.92)	0.437		2	2	0.85	(0.10 - 7.03)	0.877		7	5	1.44	(0.43 - 4.79)	0.548
Mineral Oils or Halogenated Hydrocarbons	14	4	4.52	(1.38 - 14.77)	0.012		0	0	0.87	(0.01 - 46.95)	0.946		14	4	4.13	(1.28 - 13.24)	0.017
Facial contact with equipment	14	10	1.60	(0.64 - 3.99)	0.310		2	2	0.85	(0.10 - 7.03)	0.877		16	12	1.43	(0.62 - 3.30)	0.399
Elevated Air Pollution	4	10	0.37	(0.10 - 1.25)	0.110		2	2	0.85	(0.10 - 7.03)	0.877		6	12	0.45	(0.15 - 1.28)	0.137
Continuous Mask Use	4	2	2.16	(0.37 - 12.28)	0.387		0	0	0.87	(0.01 - 46.95)	0.946		4	2	2.06	(0.36 - 11.63)	0.413
No Known Environmental Factors	3	23	0.09	(0.02 - 0.30)	<0.001		0	4	0.07	(0.00 - 1.41)	0.082		3	27	0.07	(0.02 - 0.24)	<0.001
Psychological factors																	
No depression/anxiety/stress	17	30	0.40	(0.18 - 0.87)	0.021		4	5	0.58	(0.11 - 2.88)	0.507		21	35	0.43	(0.21 - 0.85)	0.017
Depression/Anxiety/Stress	19	17	1.24	(0.56 - 2.74)	0.593		2	3	0.51	(0.07 - 3.67)	0.506		21	20	1.07	(0.51 - 2.21)	0.853
Less than 6 Hours of sleep (Sleep deprivation)	8	2	4.68	(0.94 - 23.13)	0.058		3	1	3.00	(0.27 - 33.08)	0.370		11	3	4.16	(1.10 - 15.64)	0.035
Depression/Anxiety/Stress and less than 6 hours of sleep	11	10	1.18	(0.45 - 3.03)	0.739		6	2	3.67	(0.59 - 22.78)	0.163		17	12	1.55	(0.67 - 3.54)	0.299
Diet																	
Healthy oral intake	10	27	0.25	(0.10 - 0.58)	0.001		2	6	0.18	(0.02 - 1.13)	0.068		12	33	0.04	(0.00 - 0.17)	<0.001
<2 L daily water intake	7	0	17.78	(0.99 - 319.39)	0.051		1	0	2.79	(0.10 - 74.62)	0.540		8	0	19.18	(1.08 - 339.04)	0.044
Carbohydrate Rich Diet	9	7	1.40	(0.48 - 4.05)	0.538		2	0	5.00	(0.21 - 114.22)	0.313		11	7	1.68	(0.60 - 4.61)	0.316
Oil/Salt Rich Diet	8	13	0.58	(0.21 - 1.52)	0.266		5	3	1.67	(0.31 - 8.92)	0.551		13	16	0.77	(0.33 - 1.74)	0.532
Carb/Oil/Salt Rich Diet	17	1	25.05	(3.19 - 196.26)	0.002		4	0	10.57	(0.51 - 217.75)	0.127		21	1	29.57	(3.84 - 227.25)	0.001
Carb/Oil/Salt/Dairy Rich Diet	4	9	0.42	(0.12 - 1.44)	0.169		1	4	0.16	(0.01 - 1.67)	0.127		5	13	0.34	(0.11 - 1.00)	0.051
Hormonal																	
Hormonal Imbalance	14	1	19.12	(2.41 - 151.29)	0.005		1	1	0.86	(0.04 - 15.22)	0.916		15	2	9.27	(2.03 - 42.29)	0.004
PCOS																	
Hirsutism/PCOS	12	0	33.05	(1.90 - 573.61)	0.016								12	0	33.05	(1.90 - 573.61)	0.016
Menstrual History																	
Normal Menstrual	21	54	0.03	(0.01 - 0.12 )	<0.001								21	54	0.03	(0.01 - 0.12)	<0.001
Menstrual Irregularity	23	3	12.94	(3.59 - 46.53)	<0.001								23	3	12.94	(3.59 - 46.53)	<0.001
Premenstrual Flare	3	0	7.67	(0.38 - 151.96)	0.181								3	0	7.67	(0.38 - 151.96)	0.181
Pregnancy	8	0	20.58	(1.15 - 365.86)	0.039								8	0	20.58	(1.15 - 365.86)	0.039

**Figure 1:**
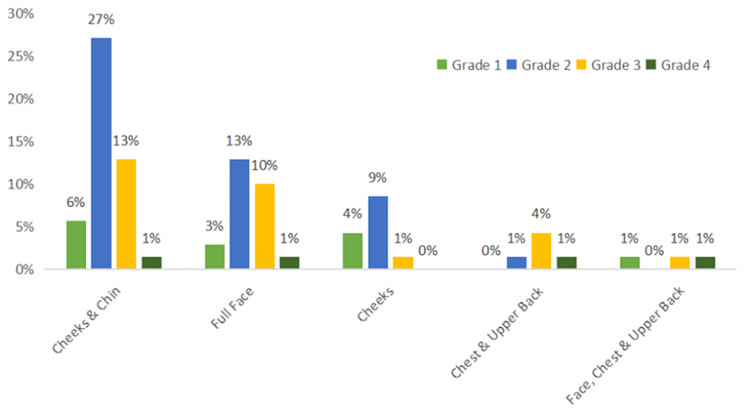
Site of acne vs. grade of acne Grade 1 Acne (light green) - represents patients who developed comedones and few papules. Grade 2 Acne (blue) - represents patients who developed papules and few pustules. Grade 3 Acne (yellow) - represents patients who developed papules, pustules, and few nodules. Grade 4 Acne (dark green) - represents patients who developed pustules, nodules, and few cysts.

The majority (47%, n=33) of patients presented with acne predominately on the cheeks and chin, followed by 27% (n=19) of patients with a full face of acne. The most prevalent grade of acne was Grade 2, seen in 50% (n=35) of patients. Late-onset adult acne was seen in 64% (n=45) of patients, persistent acne in 33% (n=22) patients, and relapse of acne was seen in 4.3% (n=3) of patients (Figure 1).

Forty-one percent (n=29) of patients had a family history of acne and 25.7% (n=18) had a previous history of adolescent or persistent acne. Having a history of acne was considered a significant factor in this study (Table 1). The OR and CI were calculated for each variable. Statistically significant findings in this subgroup included an increased odds of having AOA with a personal history of acne (OR 3.12, CI 1.20-8.03) or a family history of acne (OR 10.24, CI 2.89-36.15). No family or personal history of acne was found to be statistically significant for making it less likely to have AOA (OR 0.12, CI 0.05-0.26). Family history had the highest association with AOA.

Irregular menstrual cycle was seen in 32.9% (n=23) of patients with AOA in the case group, which was significantly higher than the control group (p=0.0002), 4.3% (n=3) of patients developed premenstrual flare-up of acne, 11% (n=8) of patients developed acne during their pregnancy. The OR and CI were calculated for each variable (Table 1). Statistically significant findings in this subgroup included an increased association with AOA with menstrual irregularity (OR 12.94, CI 3.59-46.53) and pregnancy (OR 20.58, CI 1.15 - 365.86). Normal menstrual history was found to be statistically significant for inverse association AOA (OR 0.03, CI 0.01-0.12). Pregnancy had the highest association with AOA.

Hormonal imbalance such as increased or decreased levels of LH, FSH, estrogen, testosterone, prolactin and dehydroepiandrosterone was seen in 21.4% (n=15) of patients with AOA. Seventeen percent (n=12) of females with AOA in the case group had history of polycystic ovarian syndrome (PCOS) or hirsutism which was significantly higher than the control group. The OR and CI were calculated for each variable (Table 1). Both hormonal imbalance (OR 9.27, CI 2.03-42.29) and hirsutism/PCOS (OR 33.05, CI 1.90-573.61) had a statistically significant association with AOA. Hirsutism/PCOS had the highest association with AOA.

In this study, recent or sudden weight changes were seen in 12.9% (n=9) of patients in the case group, while only 2.9% (n=2) in the control group had recent weight changes, proving significance (p=0.028). Body Mass Index (BMI) below 18 was seen in 1.4% (n=1) of patients, BMI between 18.5-24.9 was seen in 48.6% (n=34) of patients, BMI between 25-29.9 was seen in 44.3% (n=31), and 5.7% (n=4) had a BMI above 30. The OR and CI were calculated for each variable (Table 1). Statistically significant findings in this subgroup included increased odds of having AOA with an overweight BMI (OR 6.16, CI 2.56-14.76). Normal BMI was found to be statistically significantly associated with lower odds of having AOA (OR 0.18, CI 0.07-0.39). Underweight BMI and Obese BMI were not found to have any statistically significant associations in this study possibly due to less number of obese patients enrolled in our study decreasing the power of the study.

A combination of dietary factors played a more significant role in AOA than any single dietary factor. An unhealthy diet was seen in 87% (n=61) of patients. Patients were also questioned about their daily total water consumption. Fifty-three percent (n=37) of patients consumed a diet high in sugar content, trans fats, and sodium chloride (NaCl) compared following which they noticed exacerbation or new onset of acne. The OR and CI were calculated for each variable (Table 1). Statistically significant findings in this subgroup included an increased odds of having AOA with less than 2 liters of daily water intake (OR 19.18, CI 1.08-339.04), and a carbohydrate-, oil-, and salt-rich diet (OR 29.57, CI 3.84-227.25). Healthy oral intake was found to be statistically significant for making it less likely to have AOA (OR 0.04, CI 0.00-0.17). Consuming a carbohydrate-, oil-, and salt-rich diet appeared to have the highest association with AOA.

Environmental factors played a significant role in developing AOA as 95.7% (n=66) of patients were exposed to multiple risk factors, including mineral/halogenated oils, air pollution, humidity, excess sun exposure due to occupation, un-sanitized cellular phones and face masks due to the coronavirus disease 2019 (COVID-19) pandemic. The OR and CI were calculated for each variable (Table 1). Statistically significant findings in this subgroup included an increased odds of having AOA with a mineral oil or halogenated hydrocarbon use (OR 4.13, CI 1.28-13.24) and sweating/humidity/elevated temperatures (OR 3.10, CI 1.25-7.62). A report of no known environmental factors by the patient was found to be statistically significant for making it less likely to have AOA (OR 0.07, CI 0.02-0.24). Mineral oil or halogenated hydrocarbon use appears to have the highest association with AOA.

Psychological factors including stress, anxiety, depression and sleep deprivation were significantly higher among AOA patients when compared to the control group. The OR and CI were calculated for each variable (Table 1). Statistically significant findings in this subgroup included increased odds of having AOA with less than six hours of sleep (OR 4.16, CI 1.10-15.64). Having no reported issues with sleep, depression, anxiety, or stress was found to be statistically significant for making it less likely to have AOA (OR 0.43, CI 0.21-0.85). Decreased sleep appeared to have the highest association with AOA.

Of the patients that presented with AOA, 69.9% (n=49) had noticed a direct correlation between the application of cosmetic products and the development of acne. Twenty-seven percent (n=19) had an application history of topical ayurvedic products, of which 17% (n=12) developed acne after applying aloe vera-based products and face washes containing Indian lilac oil (neem oil). Salon-based procedures such as glycolic acid peels, vitamin peels, fruit facials, and laser treatment that were not done under dermatological supervision were linked to acne eruptions in 24.3% (n=17) of patients. Seventy percent of patients in the study group had some level of change in cosmetics or undergone cosmetic procedures compared with the control group. Cosmetic use was considered significant (p=0.001) in the development of adult acne. The OR and CI were calculated for each variable (Table 1). Statistically significant findings that increased odds of having AOA were only noted in females, specifically peels/facials at salons (OR 11.28, CI 2.45-51.90). Statistically significant findings associated with reduced odds of developing AOA included no associated cosmetic use (OR 0.45, CI 0.22-0.90) when combining males and females but not individually and face wash/cream/lotion use (OR 0.39, CI 0.15-0.96) for females only. A report of no associated cosmetic use by the patient was found to be statistically significant for making it less likely to have AOA (OR 0.07, CI 0.02-0.24). 

Risk factors that were not found significant in our study included the sex of the patient, occupation, history of hypertension, history of atopy, history of any immunocompromised state, alcohol, smoking, intake of dairy products and allergies to food and medicine. 

## Discussion

The mean age of AOA in the case group for this study was 30.5±5.075. Findings were consistent with Khunger et al. and Goulden et al., where the average ages of onset were 35.5 years and 30.5 years, respectively [1,2].

In this study, 64% (n=45) of patients presented with late-onset adult acne. Among all grades of acne, Grade 2 acne was predominant in 50% (n=35) patients. Forty-seven percent (n=33) of patients presented with acne on the cheeks and chin. Twenty-seven percent (n=19) of patients presented with acne over the entire face, which could be attributed to the application of various cosmetic products and beauty procedures. According to a study by Tanghetti et al., the cheeks were the most affected region on the face (79.8%), followed by the chin (77.9%) and the forehead (77.9%) [4].

In the present study, 31.4% (n=22) of patients had a positive family history of acne. Statistical analysis suggested that there is an increased likelihood of having AOA when there is a personal history of acne or a family history of acne. The results were comparable to studies conducted by Khunger et al. and Knaggs et al., where a family history of acne was present in 38.8% and 50% of patients, respectively [1,5].

The majority (70%) of patients in our study had one or more positive psychological risk factors that triggered the onset of acne. Results suggested an increased odds of having AOA if individuals who had less than six hours of sleep. Decreased sleep appeared to have the highest association with AOA. These findings were consistent with studies conducted by Goulden et al. and Poli et al. showing that psychological stress was a crucial risk factor where 71% and 58% of patients, respectively, could correlate the onset of their acne to a stressful event [2,6]. 

Environmental factors play a significant role in the development of AOA. Ninety-seven percent (n=67) of patients in the study were exposed to one or more environmental risk factors. Ten percent of AOA patients (n=7) experienced worsening acne when exposed to sunlight. In a study by Khunger et al., 33% of AOA patients experienced worsening when exposed to sunlight [1]. Twenty patients (28.5%) noticed exacerbation of acne with increased sweating and humidity. Twenty-one percent (n=15) of patients were exposed to halogenated oils, of which 4.2% (n=3) of patients developed AOA. the results suggested that individuals had an increased likelihood of having AOA with a mineral oil or halogenated hydrocarbon use as well as sweating/humidity/elevated temperatures. In a study by Shourick et al., they found that people exposed to solvent emanation, tar, oil emanation, or crude oil are more likely to develop AOA [7]. Six percent (n=4) of patients in healthcare who wore masks for more than seven hours a day for six days a week experienced mask-induced acne in our study. Dreno et al. discovered that on exposure to various environmental factors, the body responds with skin hypersensitivity, which in turn can cause exacerbation of acne [8].

In the current study, 12.8% (n=16) of patients followed skin care recommendations from social media influencers and developed acne based on their suggestions. According to Yousaf et al., 45% of adults used social media for professional acne treatment advice (54% of women vs. 31% of males), demonstrating the impact of social media skin care. Only 31% of those who used social media to make skin routine changes were compliant with the clinical criteria of the American Academy of Dermatology (AAD) [9]. Thirty-three percent (n=23) of patients had never cleaned their phones. Phones are constantly exposed to the environment and are placed on unsanitary surfaces, which makes them prone to causing facial inflammation. Upon exposure to unclean phones, patients were more prone to acne on the cheeks. Common bacterial isolates found were coagulase-negative Staphylococci (58.8%), Staphylococcus aureus (14.4%), and Klebsiella species (6.9%) [10].

Seventy percent (n=49) of the case group had applied new cosmetic products or had aesthetic procedures performed on the face before developing AOA. Procedures without dermatological advice were seen in 24.3% (n=17) of patients. Statistically significant findings included increased odds of having AOA were only noted in females, specifically peels/facials at salons and decreased likelihood of developing AOA observed with no associated cosmetic use in males and females but not individually and face wash/cream/lotion use for females only. Similarly, the use of cosmetic products led to the worsening of acne in 14.3% and 39.9% of adult females in studies conducted by Khunger et al. and Tanghetti et al. [1,4].

Multiple dietary factors played a significant role in the development of AOA. Eighty-three percent (n=58) of patients consumed a diet with an overabundance of sugar and salt compared to 52.9% (n=37) of adults in the control group. Compared to controls, acne sufferers had considerably increased NaCl consumption, suggesting that salty foods may play a role in acne development [11]. Statistically significant findings included increased odds of having AOA in individuals with less than 2 liters of daily water intake or a carbohydrate-, oil-, and salt-rich diet. Healthy oral intake was found to be associated with a decreased likelihood of AOA. A study by Darouti et al. showed that 76% of acne sufferers consumed more NaCl than recommended by the joint 'FAO/WHO expert consultation on human vitamin and mineral needs,' compared to 46.7% of control participants [12].

Kamangar et al. stated in their study that exercising, indirectly can balance hormones and decrease stress and cortisol levels, decreasing the risk of metabolic syndrome and preventing acne development [13]. In our study 44% (n=31) of patients with AOA were overweight, and 5.7% (n=4) were obese in our study. Seventy percent (n=49) had a sedentary lifestyle. Statistically significant findings included an increased odds of having AOA with an overweight BMI. Normal BMI was found to be statistically significantly associated with lower odds of having AOA.

Our current study was entirely based on patient history and clinical examination. Of the 55 female patients, 21.8% (n=12) of females had a history of PCOS or hirsutism, 41.8% (n=23) had a history of irregular menstrual cycle, and 14.5% (n=8) developed AOA during pregnancy. Both hormonal imbalance and hirsutism/PCOS had a higher likelihood of having AOA. Hirsutism/PCOS appeared to have the highest association with AOA in our study. Darley et al. found that 76% of patients with AOA had an aberrant hormonal profile [14]. Ratzer stated in his study that 29% of women reported worsening acne during pregnancy [15].

The study had a few limitations, including a small sample size and a lower number of male patients compared to female patients. On a few occasions recall bias was evident, as patients failed to remember the topical products they used in the time it took to develop acne. Furthermore, difficulty in differentiating specific dietary factors as many people were unable to accurately recall the total amount or exact content of the food that they consume per day. Additionally, social desirability bias could have affected the results as patients may have felt uncomfortable sharing information due to cultural or religious beliefs. It is important to note that the study was limited geographically to the eastern coast of India, which may have affected the findings' generalizability.

## Conclusions

AOA is a complex and multifactorial condition. This study confirmed the relevance of existing risk factors in the development of acne as well as new risk factors, such as the application of topical cosmetic products containing aloe vera and Indian lilac oil (neem oil). The factors demonstrating positive trends were positive family history of acne, overweight BMI, hormonal imbalance, menstrual irregularity in females, exposure to mineral oil or halogenated hydrocarbon use, less than six hours of sleep, chemical peels and salon-based facials and lastly diet consisting mainly of carbohydrates, high salt, saturated fats and less than 2 liters of water intake. A comprehensive approach to prevention including patient education should be provided about lifestyle modification, stress management, exercise, and environmental factors and potential side effects of salon-based procedures to help prevent and manage AOA. Specialized treatment plans according to an individual’s skin type are essential in managing AOA. Our study is one of few on AOA in the Indian skin type. This study was done under a limited demographic area in East India, where some risk factors were more prevalent than others. Further research into identifying triggers and risk factors is warranted across different demographic areas to allow for more individualized treatment plans.
